# Producing Blends of Polybutylene Adipate Terephthalate and Blood Meal That Are Safe to Render

**DOI:** 10.3390/polym15183750

**Published:** 2023-09-13

**Authors:** Casparus J. R. Verbeek, Priyal M. Yapa, Rachel Self, Mark Harrison

**Affiliations:** 1 Centre for Advanced Materials Manufacturing and Design, Faculty of Engineering, University of Auckland, Auckland 1023, New Zealand; pyap580@aucklanduni.ac.nz; 2 School of Biology and Environmental Science, Faculty of Science, Queensland University of Technology, Brisbane 4000, Australia; rachel.self@qut.edu.au (R.S.); md.harrison@qut.edu.au (M.H.)

**Keywords:** blood meal, rendering, morphology, polybutylene adipate terephthalate, polymer blends

## Abstract

Single-use plastic hygiene control products used during red meat processing can have severe negative impacts on the environment and cannot be processed with offal during rendering into meat and bone meal. However, plastics made from protein could potentially solve this problem as the material would be safe to render. The objective of this work was to prepare blends of blood meal and polybutylene adipate terephthalate (PBAT) in the absence of water using the interaction between PBAT and protein as the plasticisation mechanism. The ratio of protein to PBAT (1:1.3), as well as the choice of compatibiliser (PBAT-g-IA), was critical to form a homogenous, compatibilised blend with mechanical properties suitable for injection-moulded hygeine control products. This blend had a tensile strenght of 11.2 MPa, a chord modulus of 492 MPa, and 10% elongation at break. Using less PBAT in the blend, or using Surlyn™ as a compatibiliser, resulted in blends that were either too difficult to process or with inferior mechancial properies. Using simulated rendering, the new material was indistinguishable from tallow or meat and bone meal, suggesting that hygeine control products made from this new material will degrade sufficiently to be safe to render with offal after red meat processing.

## 1. Introduction

Single-use plastics have severe negative impacts on the environment and take hundreds of years to degrade, resulting in harmful pollution to ecosystems and wildlife [1]. Bio-based materials manufactured from renewable polymers such as soy protein, starch, or gluten offer the opportunity to reduce plastic pollution and resource depletion [2,3]. However, renewable thermoplastics, such as those from proteins, typically have relatively high glass transition temperatures (T_g_), leading to difficulties in processing [4]. To improve the mechanical properties and processability of protein-based thermoplastics, native proteins are typically denatured with heat and additives (such as urea, sodium dodecyl sulphate, and sodium sulphite) are included to facilitate protein unfolding. Protein unfolding allows the formation of intermolecular interactions in the presence of water and plasticisers, and this process generally leads to the formation of a thermoplastic protein [5,6,7]. Alternatively, protein-based thermoplastics may also be blended with other polymers to improve mechanical or physical properties, especially resistance to water when used in humid environments.

Polybutylene adipate terephthalate (PBAT) is a biodegradable polymer and has become readily available. Although not currently bioderived, PBAT is less expensive than other biopolymers and, therefore, economically attractive for incorporation into polymer blends. PBAT is a random copolymer, which means that it cannot crystallise significantly and thus, has a relatively wide melting point, low elastic modulus, and stiffness, but has relatively high flexibility and toughness, making it ideal for improving the toughness of polymer blends. Because of these properties, PBAT is often blended with protein-based thermoplastics (e.g., thermoplastic whey [4,8], soy [9,10,11,12], zein [13], sodium caseinate [14], and blood meal [15,16]), and a compatibiliser is typically included in these blends to aid in interfacial adhesion. Common compatibilisers are maleic anhydride-grafted PBAT [10,11], poly-2-ethyl-2-oxazoline (PEOX), pMDI [17], itaconic anhydride-grafted PBAT [16], poly(ethylene glycol) diglycidyl ether) [13], and glycidyl methacrylate-grafted PBAT. When forming a compatible blend, the compatibiliser promotes entanglement between polymer chains which increases T_g_, cold crystallisation temperature and melting temperature [18].

A key challenge in producing thermoplastic protein blends with PBAT is the formation of a homogenous material. Many studies have found that protein forms a dispersed phase in PBAT [8,9,10,11,13]. In almost all these examples, the protein fraction still required plasticisation (e.g., with glycerol, triethylene glycol etc.) in the presence or absence of water. The plasticiser aids in the rearrangement of polymer chains, leading to the formation of a homogenous blend [4,11,19].

The use of water and other plasticisers should be avoided when blending thermoplastic protein with other polymers. Using water during compounding ensures that the protein behaves like a plastic. However, once most of the water has evaporated after drying, plastic flow becomes almost impossible; thus, these materials are then often thought of as composites [19]. Forming a blend without water as a processing aid still requires a compatibiliser. For example, blending zein with PBAT and using poly(ethylene glycol) diglycidyl ether as a compatibiliser led to a fine dispersion of zein in the PBAT matrix with good mechanical properties [13]; however, without a compatibiliser the protein simply acted as a filler [20].

Red meat processing facilities use plastic hygeine control products to seal the intestinal tract during processing and these plastics can end up in landfill [21]. Polypropylene and polyacetal are currently used to make these hygeine control products but they are not safe to render. Rendering transforms red meat processing by-products into meat-and-bone meal, blood meal, and tallow, and processing aids made from polypropylene and polyacetal can contaminate these important products. Novatein^®^ is a newly developed material made from blood meal, a by-product of the red meat processing industry with high protein content [17]. Novatein^®^ is a biodegradable, thermoplastic protein but it becomes brittle over time due to loss of plasticiser; however, this can be mitigated by blending with other polymers [16], similar to many other protein-based materials [4,18,22]. Novatein^®^ is best suited for agricultural and horticultural applications, but it lacks the durability required for hygeine control products in red meat processing.

The production of a protein-based material suitable for application in hygiene control during red meat processing necessitated finding a balance between moisture resistance (to preserve mechanical properties during use), safety during rendering, and mechanical characteristics. The objective of this work was to produce a protein-based polymer in the absence of water or any other plasticiser and aimed to achieve sufficient ductility without compromising tensile strength. Building on previous work, modified blood meal powder was blended with PBAT and two different compatibilisers without any other plasticisers. The suitability of the resulting material for rendering was evaluated in a simulated low-temperature rendering process.

## 2. Materials and Methods

### 2.1. Materials

PBAT was sourced from Avient (Auckland, New Zealand), under the trade name Renol 03459 PBAT. Surlyn 9320 (SUR) is a zinc ionomer thermoplastic resin, otherwise described as an ethylene/acid/acylate terpolymer in which some of the methacrylic acid groups have been partially neutralised with zinc oxide. Surlyn™ has a melt flow index (MFI, 2.16 kg, 190 °C) of 0.8 g·10 min^−1^; it was produced by DuPont and acquired through IMCD, Auckland, New Zealand. Reagent grade itaconic anhydride (IA), dicumyl peroxide (DCP), sodium dodecyl sulphate (SDS) and sodium sulphite (SS) were procured from Sigma-Aldrich, St. Louis, MO, USA. Blood meal was obtained from Hawkes Bay protein, Napier, New Zealand, and contained ~5 wt.% moisture.

### 2.2. Methods

#### 2.2.1. Grafting

PBAT pellets were dried overnight in a Moretto drier (Massanzago, Padua, Italy) at 60 °C for 24 h. IA and DCP powders were mixed at 0.5% and 0.4% wt.% of PBAT prior to extrusion. The formulation required a dual extrusion process on a Lab-Tech twin-screw extruder (L/D = 44). The first extrusion included the addition of the mixed IA and DCP dosed into the final mixing zone of the extruder. A screw speed of 180 rpm was used with the following temperature profile: 140 °C for zone one, 150 °C for zones two–five, 160 °C for zones six and seven, 150 °C for zones eight and nine, and 140 °C for the die. The extrudate was then pelletised and re-extruded at an increased screw speed of 250 rpm and a temperature profile of 150 °C for zone one, 170 °C for zones two–five, 180 °C for zones six through to nine, and 170 °C for the die.

#### 2.2.2. Compounding

Components were dried for 24 h at 60 °C for all blends. Three different blends were prepared by blending modified protein powder (blood meal plus SDS and SS, proprietary blend) with either 100 or 130 parts PBAT per 100 parts modified protein (Table 1). The approximate amount of PBAT and compatibiliser was determined after initial scoping trials. The 100-parts PBAT blends were compatibilised using either IA-g-PBAT or Surlyn™ by replacing the required fraction of PBAT with compatibiliser. All blends were prepared at a commercial facility using a Sino Alloy twin screw extruder (L/D = 40 with a 50 mm screw diameter) from Taoyuan City, Taiwan. The temperature profile used for extrusion started with the feed region of 70 °C, 150 °C for zones one–five, 145 °C for zones six and seven, and 130 °C for zone eight and the die. The extrudate was water cooled and pelletised with an inline cutter.

#### 2.2.3. Injection Moulding

A Boy 50A injection moulder was used to fabricate tensile (ASTM D638 Type 1 [23]) and impact (ASTM D6110 [24]) specimens. Material was injection moulded at 150 °C into a water-cooled mould.

#### 2.2.4. Mechanical Testing

An Instron 5567 universal testing machine with a 30 kN load cell was used to determine tensile properties of all materials. Blends were tested at a crosshead speed of 5 mm·min^−1^ for blends and 300 mm·min^−1^ for PBAT and IA-gPBAT, respectively. All specimens had a grip distance of 115 mm and a gauge length of 50 mm. The chord modulus was determined between 0.05% and 0.25% strain. Impact strength was determined according to ASTM D6110 using a 0.5 J hammer. All mechanical properties were measured with no fewer than five repetitions.

#### 2.2.5. Thermal Properties

Thermal properties were determined using a TA Instruments DSC Q1000 differential scanning calorimeter (DSC) and TA Instruments DMA Q800 dynamic mechanical analysis (DMA) instrument (New Castle, DE, USA). Both devices were calibrated according to the manufacturer’s processes. DSC measurements were determined through a heat-cool-heat process with a heating rate of 10 °C·min^−1^ and a nitrogen flow rate of 50 mL·min^−1^ over a sealed aluminium pan. Samples were taken from room temperature to 200 °C, then cooled to −50 °C and held for 2 min, before reheating up to 250 °C. DMA measurements were taken using a single cantilever configuration, with specimen dimensions of 17.5 mm × 12.6 mm × 3.0 mm. A dynamic displacement of 0.05 mm and a heating rate of 2 °C·min^−1^ were used between −80 °C and 120 °C. The specimens were subjected to six oscillation frequencies: 0.1, 0.3, 1.0, 3.0, 10, and 30 Hz.

#### 2.2.6. Water Absorption

Water absorption was measured by soaking in an Elma D-78224 Singen/Htw ultrasonic heated water bath at 37 °C over the course of 2 h (Singen, Germany). Water uptake was determined at intervals of 1, 2, 5, 15, 30, 60, 90, and 120 min. The temperature selected was to best match operational conditions in a red meat processing facility, as described in the introduction.

#### 2.2.7. Morphology

An FEI Philips XL30 S-FEG scanning electron microscope (SEM) operated (Hillsboro, OR, USA) at 10 kV was used to evaluate the morphology of cryo-fractured injection-moulded specimens. Specimens were cryo-fractured in liquid nitrogen and the cross-sectional fracture surfaces were double sputter-coated in platinum.

#### 2.2.8. Simulated Low Temperature Rendering

Commercial rendering is undertaken as a continuous or batch process either in the presence of excess water (‘low-temperature’ rendering) or after the water in the render has been evaporated (‘high-temperature’ rendering). The low temperature rendering process begins with particle size reduction to ~12 mm and heating to ~95 °C for 5–60 min. The liquid and solid in the heated slurry are separated via a screw press or centrifuge, and the liquid is separated into water and fat (tallow) fractions using a second centrifugation step. The solid (which contains residual water) is dried for 60–120 min using an indirect steam heated drier with the end point temperature that does not exceed 110 °C. The dried solids are then milled to produce a free-flowing meal. As a result, the components of any extraneous matter (e.g., plastic) in the render partition between three phases (i.e., fat, water, and solid) during low temperature rendering.

Animal co-products used in the rendering process typically include inedible offal, bones, and fat. To simulate rendering products at laboratory-scale, commercially available meat products were prepared including raw pet meat (particle size ~10 mm), sheep liver, and fatty beef soup bones. Sheep liver was chopped into ~10 mm cubes, and scraps of beef meat and fat were removed from the beef bones (~0.5–2.0 mm particles). Beef bones were cooked at 120 °C for 3 h to soften and then size reduced using a Waring commercial blender.

Pellets of bioplastic (Blend IA13) were milled using a Retsch Knife Mill fitted with a 2 mm screen and blended with animal co-products at 0.1, 1, and 5 wt.%. MBM was added to animal co-products in the same ratio without any further size reduction.The average amount of hygiene products in low temperature rendering of co-products from beef cattle is <0.01 wt.%; thus, the selected inclusion rates in the present study are 10, 100, and 500 times higher than would be expected in red meat processing. Blends of animal co-products and the bioplastics were subjected to simulated low temperature rendering using a laboratory autoclave set to minimum temperature (105 °C) for 20 min. After heating, larger solids were separated from liquid (tallow and water) through a conical sieve and the remaining solids and liquid phases were separated by centrifugation at 3500 rpm for 20 min. The solids were dried at 105 °C for 3 h and milled into meat and bone meal (MBM) using an Ika Mill fitted with a 2 mm screen. The liquid phases were stored at 4 °C overnight to solidify the tallow layer, which was recovered manually and dried overnight at 60 °C.

#### 2.2.9. Fourier-Transform Infrared Spectroscopy (FT-IR)

(FT-IR) analysis was performed on products from rendering offal plus MBM, modified protein as well as IA13, using a Nicolet iS50 FT-IR spectrometer, Thermo-Fisher, Brisbane, Australia. Spectra were taken of the solids remaining after drying as well as the separated fat (tallow). The average of three representative spectra for each sample, taken between 4000 and 400 wavenumber.cm^−1^, were selected and corrected for attenuated total reflection.

## 3. Results and Discussion

### 3.1. Mechanical Properties

**Figure 1:**
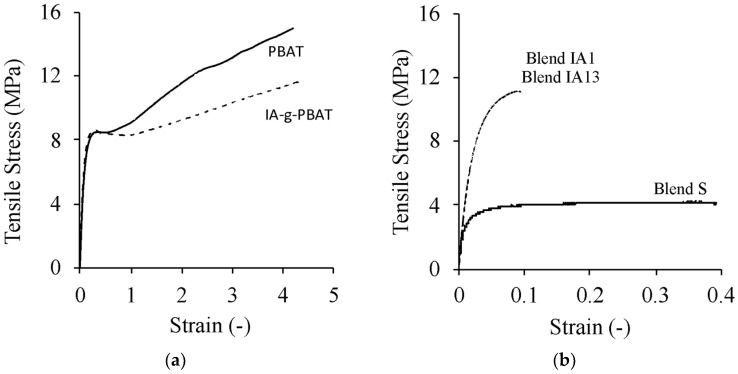
Representative stress-strain graphs for (**a**) PBAT and IA-g-PBAT and (**b**) Blends S, IA1 and IA3.

Blends were prepared using a significant amount of IA-g-PBAT (Table 1); therefore, the changes in thermal and mechanical properties of PBAT resulting from IA grafting were evaluated (Table 2 and Table 3). Grafting significantly altered the mechanical properties of PBAT; it increased modulus and lowered the ultimate tensile stress (UTS). This was most likely due to unwanted side reactions during grafting, such as crosslinking and chain scission [25]. Interestingly, the strain at break was largely unaffected, and both PBAT and IA-g-PBAT yielded at approximately the same strain (8.6 MPa yield strength) (Figure 1a). Comparison of stress-strain for PBAT and IA-g-PBAT revealed that PBAT strain hardened more than the grafted copolymer (Figure 1a).

**Figure 2:**
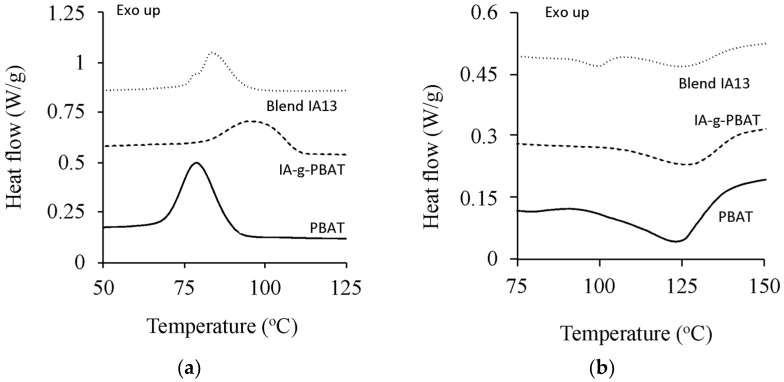
(**a**) DSC cooling and (**b**) DSC heating thermograms as tested using DSC, showing crystallisation and melting behaviour.

**Figure 3:**
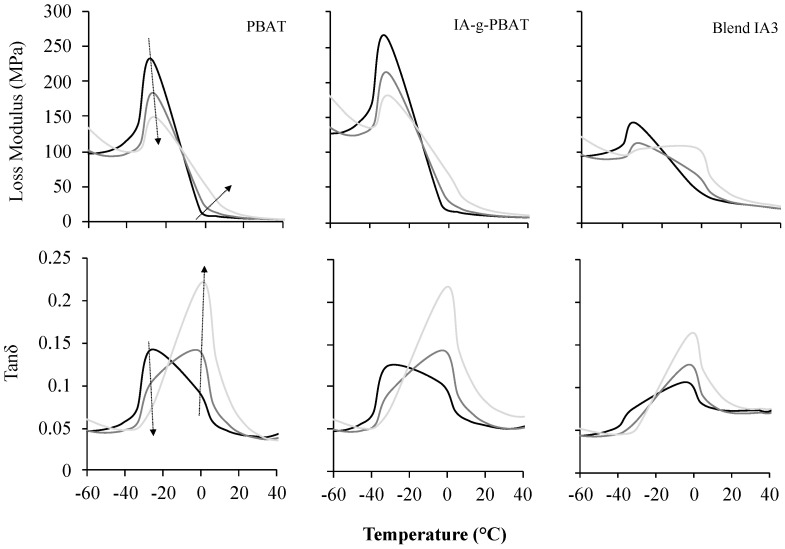
Loss modulus and Tan δ as a function of temperature, showing the glass transition temperature (peak in Tan δ or loss modulus), as measured using dynamic mechanical analysis. Arrows indicate increasing frequency; 0.1, 1 and 30 Hz.

The grafting of IA onto PBAT increased cold crystallisation temperature from 79.4 to 97.7 °C (Figure 2). A higher crystallisation temperature suggests reduced chain mobility, which could be from the bulkier substituent (IA) or some degree of crosslinking. From previous work, however, crosslinking should be minimal at the initiator and monomer concentrations used during grafting [16]. The latent heat of crystallisation decreased by 45% (26.5 J·g^−1^ to 14.7 J·g^−1^), suggesting IA-g-PBAT was less crystalline than PBAT and consistent with disruption of regular, close packing of the chains in the presence of IA. The melting point did not undergo such substanial changes, with an average increase of 3.6 °C, but the energy required to melt was reduced by just over 50% (Table 3). From DMA, only minor differences were observed between IA-g-PBAT and PBAT at 1 Hz. A subtle shoulder region appeared in the tan δ curve for the copolymer (Figure 3), suggesting some minor variation in chain structure between these polymers.

Tailoring the mechanical properties of blends of modified protein and PBAT depends on the degree of compatibility and the morphology of the blend [22]. Three variants were considered (Table 1). By altering either the amount of protein or the type of compatibiliser, we expected significant changes to the mechanical properties of the blend. Using IA-g-PBAT as a compatibiliser produced the best results for UTS and impact strength. At low PBAT content (Blend IA1), the modulus and UTS were the highest, but strain at break and impact strength was lower. Considering high strength (including impact) with a high strain at break are desirable properties, we were faced with a challenge; increasing the proportion of PBAT would reduce the bio-based content in the blend while decreasing the proportion of PBAT below 100 would prevent injection moulding because of high viscosity.

The additon of Surlyn™ as a compatibiliser resulted in much higher strain at break, lower UTS (3.68 MPa), and a comparable modulus to IA-g-PBAT. The impact strength was the lowest of the three blends (55.3 J·m^−1^), significantly lower than Blend IA13, which in total contained the same amount of non-proteinaceous material. The difference between these blends therefore rested in the degree of compatibility, as was observed through SEM.

### 3.2. Morphology

**Figure 4:**
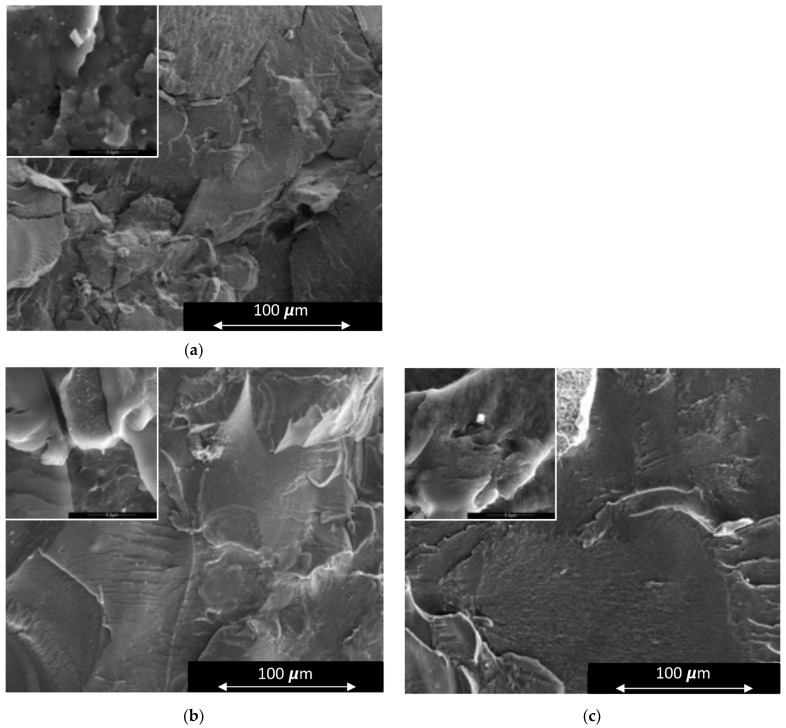
SEM images of (**a**) Blend S, (**b**) Blend IA1 and (**c**) Blend IA13.

The morphology of the Surlyn™ blend (Blend S) showed clear signs of non-homogeneity (Figure 4). Differing phases of PBAT and protein were distinctly visible in the fracture surfaces, as were spherical Surlyn™ particles. This demonstrated that using Surlyn™ as a compatibiliser was ineffective and led to weakly bonded protein-rich regions dispersed amongst PBAT. As a result, under deformation, the PBAT was relatively unconstrained, and the blend showed a very high strain at break. Despite the high strain at break, the low strength of this blend would make it unsuitable for products under load, such as hygeine control products in red meat processing.

The distinct two-phase system between blood meal and PBAT was not present in the two IA-g-PBAT blends (Blends IA1 and IA13) (Figure 4). The addition of the copolymer resulted in an almost indistinguishable phase structure between PBAT and protein (minor phase separation was visible at higher magnification). The anhydride functional group is much more reactive towards protein (compared to unfunctionalized PBAT), forming strong bonds between the protein chains and IA-g-PBAT. It is worth noting that in this work, no water (or other plasticiser) was used as part of the protein-based fraction. It is common practise to include a significant amount of water and plasticisers when processing protein-based plastics, as seen in many studies for plant or animal derived protein [4,7,10]. These are normally required to assist in chain rearrangement during processing, which is vital for the formation of a cohesive material. In previous work, when blood meal was used as the protein source and blended with PBAT, the protein fraction was first converted to a thermoplastic before blending with PBAT [16]. Even though this was successful, the presence of water during processes like injection moulding was problematic and led to excessive shrinkage, and the morphology of these blends were more comparable to that of the Surlyn™ blends in this work. The implication is that protein chain rearrangement can arise solely from the interaction with synthetic polymers with reactive functional groups, making it possible to produce protein-based thermoplastics in the absence of water and other plasticisers. For this reason, only the 130-PBAT blend was evaluated in the latter part of this study.

### 3.3. Thermal Properties

**Figure 5:**
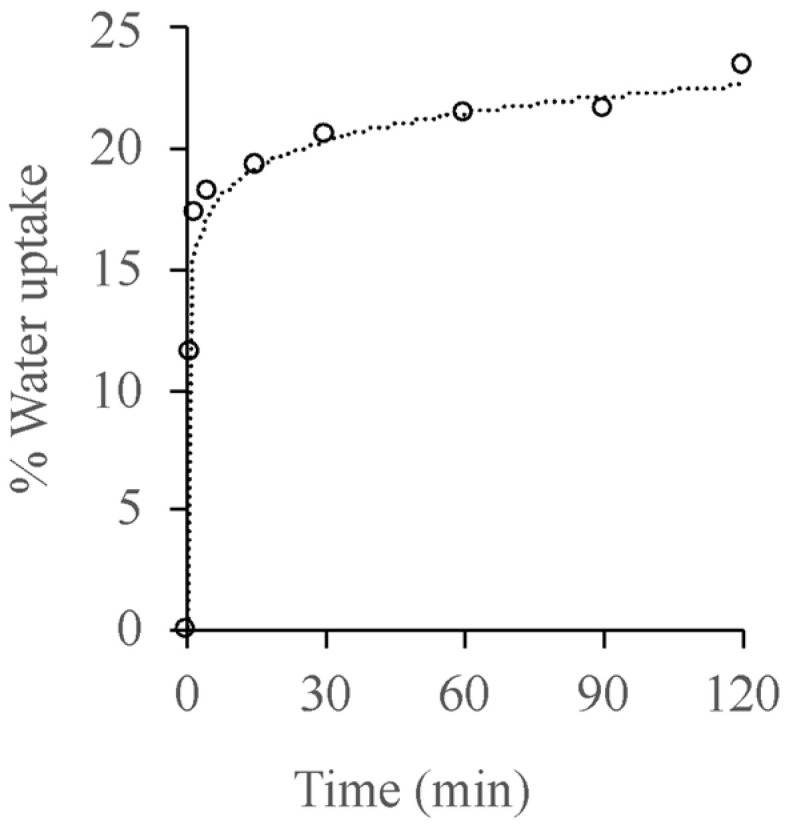
Rate of water absorption for Blend IA13.

In contrast to PBAT and the copolymer, the thermal behaviour of Blend IA13 included a second, lower crystallisation temperature, as well as two distinct melting points (Figure 5). From the cooling curve (Figure 5), the combined latent heat was 10.7 J·g^−1^, less than the PBAT fractions in the blend (13.1 J·g^−1^). The second peak was at 79 °C, identical to that of virgin PBAT. In the blend, crystallisation was hampered by the presence of the protein fraction, which strongly bound to the PBAT phase (Figure 2c).

Blend IA13 melting occurred in two stages (Figure 2c), which was distinctly different to IA-g-PBAT and PBAT. The highest peak was at 127 °C, approximately that of PBAT, while the lower melting point was at 100 °C. The latent heat of fusion for these fractions were 0.83 J·g^−1^ and 3.30 J·g^−1^ respectively, and lower than what would be expected from the PBAT fractions of the blend (6.6 J·g^−1^) (Table 3). This would suggest that not all the available PBAT could crystallise because of the interference of the amorphous protein chains. Further, a small fraction of PBAT melting at a lower temperature was likely due to smaller, imperfect crystals, compared to the bulk PBAT fraction.

Thermal behaviour was further explored using DMA and measured by loss modulus and tan δ (Figure 4). A clear peak at about −20 °C was observed for PBAT and IA-g-PBAT, commonly taken as the T_g_. The T_g_ of the copolymer wasn’t significantly lower than that of virgin PBAT. However, in both cases, as the frequency was increased to 30 Hz, a shoulder region appeared at about 0 °C and was slightly more pronounced for the copolymer. This behaviour was more apparent from the tan δ curves, where (at higher frequency) the T_g_ clearly shifted to a higher temperature. In this case, the polymer chains do not have sufficient time to relax during deformation and a higher T_g_ was observed, although the T_g_ region was rather broad and the lower temperature T_g_ did remain (albeit less pronounced). This could be indicative of variability in chain composition and mobility, as would be expected from PBAT (PBAT is also a copolymer where the aromatic portion can have varying chain lengths).

However, considering Blend IA13, the loss modulus had a much more pronounced shoulder region (as seen in IA-g-PBAT) at higher frequency. It was also evident from the tan δ curves that the T_g_ for the blend was much closer to 0 °C even at lower frequency. This observation suggests that chain movement was severely restricted in the compatibilised blend.

### 3.4. Water Absorption

When submerged, Blend IA13 rapidly absorbed water and reached a plateau of ~23% water uptake after 2 h (Figure 5). Only Blend IA13 was evaluated as the other blends were dismissed as candidates based on their processability and mechanical properties. Previous work has shown that blood meal-based thermoplastics absorb more than 100% water (similar to other protein-based materials [7]), although the degree of water absorption was reduced to similar levels after blending with polyethylene [17]. PBAT was not tested for water absorption as it is a hydrophobic polymer with negligible water absorption (less than 1 wt.% over 20 h) [26]. This would suggest that the blend is still reasonably hydrophilic, which should aid in breakdown during rendering. After 2 h of submersion in water, the UTS and modulus decreased by 5.5% and 16.6%, respectively, while the strain at break increased by 65%. These relatively small changes after water absorption would make products from this material suitable for using in a moist environment, such as hygiene control during red meat processing.

### 3.5. Simulated Low Temperature Rendering

MBM was produced from simulated low temperature rendering of animal co-products with modified blood meal powder blends added at 0.1, 1, and 5 wt.%. Samples were dried and milled through a 2 mm screen to produce a free-flowing meal, and visually compared. A sub-sample of each MBM and tallow sample was analysed by FT-IR to identify spectral features associated with the presence of modified protein or blends with PBAT in the samples. In addition, samples of modified protein, Blends IA13, MBM, and tallow were analysed to provide a baseline for comparisons. The characteristic peaks of PBAT can be defined around 3000 cm^−1^ (C–H stretching), 1710 cm^−1^ (C=O in ester linkages), 1260 cm^−1^ (C-O in ester linkages), and at 720 cm^−1^ (-CH_2_-). For MBM, the most characteristics peaks are the amide I (1600–1800 cm^–1^), II (1470–1570 cm^–1^), and III (1250–1350 cm^–1^) regions.

**Figure 6:**
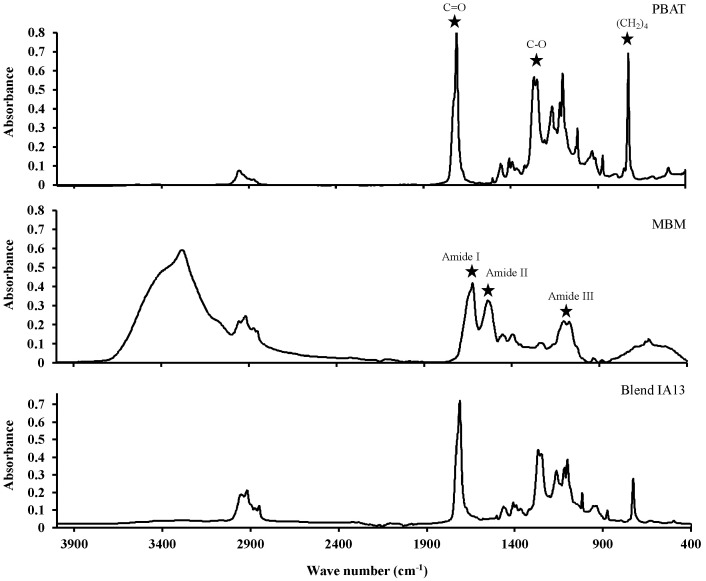
FTIR spectra of PBAT, meat and bone meal and Blend IA13. Samples were analysed in triplicate, the average representative spectra chosen and data ATR corrected. Characteristic peaks are labelled.

**Figure 7:**
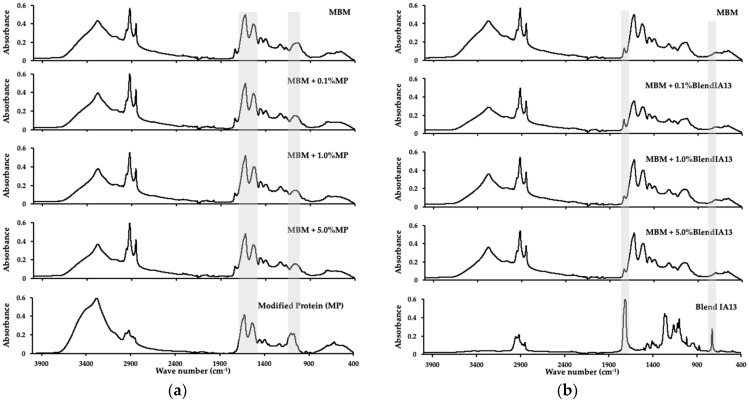
FTIR spectra of meat and bone meal containing 0, 0.1, 1, and 5% modified protein powder (**a**) and 0, 0.1, 1, and 5% Blend IA13 (**b**). Samples were analysed in triplicate, the average representative spectra chosen, and data ATR corrected. The highlighted regions show the important areas where differences may be expected.

**Figure 8:**
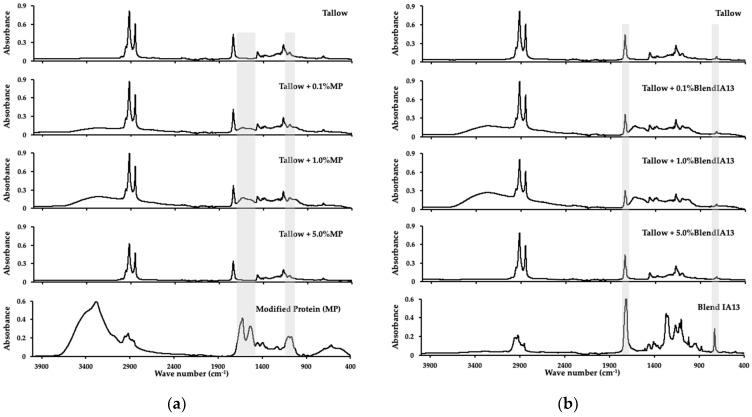
FTIR spectra of tallow samples containing 0, 0.1, 1, and 5% modified protein (**a**) and 0, 0.1, 1, and 5% Blend IA13 (**b**). Samples were analysed in triplicate, the average representative spectra chosen, and data ATR corrected. The highlighted regions show the important areas where differences may be expected.

There was no observable difference in visual appearance between MBM, and MBM containing either 0.1 or 1 wt.% of Blend IA13. The FT-IR spectra of modified protein and Blend IA13 (Figure 6) were noticeably different, with the only prominent similarity being the group of four peaks around 2900 wave number, a region of the FT-IR spectrum typically associated with -CH stretching. The FT-IR spectra of MBM samples containing Blend IA13 (Figure 7) were similar to MBM containing modified protein at all inclusion rates. The broad spectral feature between 3000 and 3750 wave numbers indicated the presence of bound water in the sample, and thus, was not diagnostic of modified protein being detected in the MBM samples. Blend IA13 was difficult to detect in FT-IR spectra (Figure 7) of MBM at inclusion rates of 5% or lower. The FT-IR spectra of tallow samples from wet rendering in the presence of either modified protein or blends thereof did not contain any significant peaks associated with the presence of bioplastic (Figure 8). It was therefore concluded that hygiene products for red meat processing made from this blend would be safe to render.

## 4. Conclusions

In this study, blends of modified blood meal powder with PBAT were prepared in the absence of water, which is normally required for processing protein-based thermoplastics. IA-g-PBAT provided superior interaction with the protein over Surlyn™ and led to sufficient plasticisation. It was found compatibilising the blend with IA-g-PBAT led to a homogenous microstructure rather than the filler-matrix structure observed when using Surlyn™. Although decreasing the proportion of PBAT in the blend slightly increased the tensile strength, a ratio of 1:1.3 was considered optimal in light of a higher impact strength and elongation at break. Using a higher proprtion of PBAT as a plasticiser also made injection moulding easier.

Including PBAT in the blends did not compromise the ability of the material to be rendered along with other offal into meat and bone meal. It was found that PBAT and blood meal degraded sufficiently to be indistinguishable from either tallow or meat and bone meal, both important products from rendering. Products made from this new material would therefore be suitable for hygiene control during red meat processing and will not require sepration from offal before rendering, leading to significant cost savings for renderers.

## Figures and Tables

**Table 1:** Formulations used for compounding blends with modified protein.

	Modified Protein	PBAT	IA-g-PBAT	Surlyn
	(Parts)			
Blend S	100	104	0	26
Blend IA1	100	80	20	0
Blend IA13	100	104	26	0

**Table 2:** Mechanical properties of polymers and blends with modified protein. Values in brackets are the standard deviation from 5 samples. PBAT and IA-g-PBAT did not break under impact.

	Chord Modulus (MPa)	UTS ^1^ (Mpa)	∑_b_ (−) ^2^	Impact Strength (J·m^−1^)
Neat polymers				
PBAT	46.6 (±1.0)	15.1 (±0.23)	4.2 (±0.009)	-
IA-g-PBAT	93.1 (± 3.8)	11.6 (±0.17)	4.3 (±0.008)	-
Polymer blends				
Blend S	494 (±70.0)	3.68 (±0.26)	0.37 (±0.063)	55.3 (±4.5)
Blend IA1	647 (±70.3)	11.6 (±0.06)	0.07 (±0.007)	58.5 (±1.9)
Blend IA13	492 (±28.6)	11.2 (±0.08)	0.1 (±0.008)	68.9 (±4.9)

^1^ Ultimate tensile strength. ^2^ Strain at break.

**Table 3:** Thermal properties. as tested using DSC, showing crystallisation and melting behaviour.

	Cooling		Heating	
	T_c_ (°C)	ΔH_c_ (J·g^−1^)	T_m_ (°C)	ΔH_m_ (J·g^−1^)
PBAT	79.4	26.5	124.9	13.7
IA-g-PBAT	97.7	14.7	128.6	6.53
Blend IA13	79.0/83.4	10.7	99.9/127.3	0.83/3.3

## Data Availability

Data is contained within the article.
